# Comparison of human and mouse E-selectin binding to Sialyl-Lewis^x^

**DOI:** 10.1186/s12900-016-0060-x

**Published:** 2016-07-02

**Authors:** Anne D. Rocheleau, Thong M. Cao, Tait Takitani, Michael R. King

**Affiliations:** Meinig School of Biomedical Engineering, Cornell University, Ithaca, NY USA

**Keywords:** E-selectin, Receptor, Cell adhesion, Molecular dynamics, Docking, Steered molecular dynamics

## Abstract

**Background:**

During inflammation, leukocytes are captured by the selectin family of adhesion receptors lining blood vessels to facilitate exit from the bloodstream. E-selectin is upregulated on stimulated endothelial cells and binds to several ligands on the surface of leukocytes. Selectin:ligand interactions are mediated in part by the interaction between the lectin domain and Sialyl-Lewis x (sLe^x^), a tetrasaccharide common to selectin ligands. There is a high degree of homology between selectins of various species: about 72 and 60 % in the lectin and EGF domains, respectively. In this study, molecular dynamics, docking, and steered molecular dynamics simulations were used to compare the binding and dissociation mechanisms of sLe^x^ with mouse and human E-selectin. First, a mouse E-selectin homology model was generated using the human E-selectin crystal structure as a template.

**Results:**

Mouse E-selectin was found to have a greater interdomain angle, which has been previously shown to correlate with stronger binding among selectins. sLe^x^ was docked onto human and mouse E-selectin, and the mouse complex was found to have a higher free energy of binding and a lower dissociation constant, suggesting stronger binding. The mouse complex had higher flexibility in a few key residues. Finally, steered molecular dynamics was used to dissociate the complexes at force loading rates of 2000–5000 pm/ps^2^. The mouse complex took longer to dissociate at every force loading rate and the difference was statistically significant at 3000 pm/ps^2^. When sLe^x^-coated microspheres were perfused through microtubes coated with human or mouse E-selectin, the particles rolled more slowly on mouse E-selectin.

**Conclusions:**

Both molecular dynamics simulations and microsphere adhesion experiments show that mouse E-selectin protein binds more strongly to sialyl Lewis x ligand than human E-selectin. This difference was explained by a greater interdomain angle for mouse E-selectin, and greater flexibility in key residues. Future work could introduce similar amino acid substitutions into the human E-selectin sequence to further modulate adhesion behavior.

## Background

Selectins are a family of transmembrane adhesion molecules that mediate the inflammatory response and the cancer metastasis cascade. There are three members of the selectin family: P(latelet)-selectin, E(ndothelial)-selectin, and L(eukocyte)-selectin. All three contain an N-terminal lectin domain, epidermal-growth-factor-like (EGF) domain, a varying number of consensus repeat units, a transmembrane portion, and a cytoplasmic tail [1–3]. During inflammation, fast binding and dissociation of bonds between cells and endothelium contributes to rolling. Selectin:ligand interactions are mediated partially by the interaction between the lectin domain and Sialyl Lewis x (sLe^x^), a tetra saccharide on cell surface proteins common to selectin ligands. E-selectin binds particularly well to PSGL-1, CD44, and ESL-1 [1, 4].

**Fig. 1:**
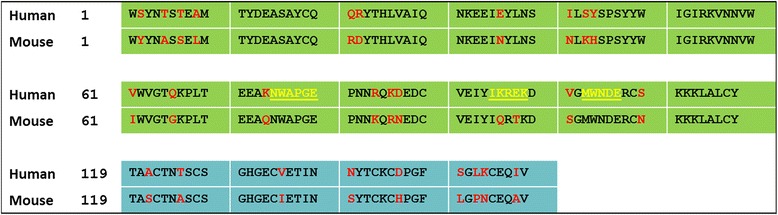
Sequence alignment of EGF and lectin domains of human and mouse E-selectin. The lectin domain is shown in green, and the EGF domain is shown in teal. Residue differences between species are noted in red, and the binding pocket for human E-selectin is noted in yellow and underlined

There is a high degree of amino acid identity between selectins of various species: about 72 and 60 % in the lectin and EGF domains, respectively [3]. Mouse E-selectin differs from human E-selectin by 29 substitutions in the lectin and EGF domains (Fig. 1). The amino acid differences between human and mouse E-selectin are fairly evenly distributed within and between the domains (Fig. 1).

Molecule conformational changes are essential to physiological processes [5]. Selectin interdomain hinge flexibility greatly affects the on-rate of selectin:ligand binding. All the selectins have shown “open” and “closed” states that correspond to whether or not they are in complex; for instance, there is a 52° increase in the interdomain angle from unliganded P-selectin to P-selectin in complex [6]. Hydrodynamic forces in the bloodstream favor the open conformation as it can strengthen selectin:ligand bonds [7]. A flexible hinge encourages the oscillation between the two states, which facilitates greater range of motion for the lectin domain and thus provides more opportunity for binding [8, 9]. Lou et al. used molecular dynamics (MD) and site mutagenesis at the interdomain hinge of L-selectin to learn that increasing hinge flexibility via mutation caused an increase in binding on- and off-rates of selectin:ligand interactions [10]. Of particular interest are the binding site and interdomain angle, since prior dissociation studies of P-selectin:sLe^x^ suggest these to be important modulators of dissociation time and final conformation [11].

MD simulations are a useful tool to study the movement of a protein chain over time, given specified starting parameters [12]. The goal of this study was to determine how the structural differences between human and mouse E-selectin affect their corresponding binding and thus cell rolling behavior. MD, docking, and steered molecular dynamics (SMD) were used in conjugation with microtube rolling experiments to address this link between molecular properties and cellular scale adhesion phenomena under flow.

## Methods

### MD to prepare receptor (E-selectin or mutants) for docking

The lectin and EGF crystal structure of human E-selectin (1ESL) was obtained from the Protein Data Bank to provide starting atomic coordinates. The lectin and EGF domains are the effective binding unit of E-selectin. The E-selectin:sLe^x^ complex crystal structure (1G1T) was not used as a starting structure as the bound complex does not allow for full flexibility of E-selectin when amino acid substitutions are made. MD, docking, and SMD simulations were performed using the YASARA (YASARA Biosciences GmbH, Vienna, Austria) package of MD programs with the YAMBER3 self-parameterizing force field. For all simulations, the temperature and pressure were held constant at 298 K and 1 atm, respectively. Other parameters used include periodic boundary conditions, the particle mesh Ewald method for electrostatic interactions, and the recommended 7.86 Å force cutoff for long-range interactions [13]. A predicted model of mouse E-selectin was created using human E-selectin as a template and substituting 29 residues.

For equilibrium simulations, human and mouse E-selectin were each solvated in a water box and neutralized by adding Na^+^ and Cl^-^ ions to a concentration of ~50 mM. To allow for free protein rotation, the water box was defined as a cube with sides 80 Å, at least 10 Å from the structure. The conformational stresses were removed using short steepest-descent minimizations followed by simulated annealing until sufficient convergences were reached. Free dynamics simulations were run for 10 ns. Similar equilibration simulations were run for sLe^x^ (taken from the 1G1T PDB structure) with a water box of size 30 × 30 × 30 Å. The average structure for each simulation run was used for further simulation steps.

### Binding sLe^x^ to human and mouse E-selectin

Molecular docking predicts the conformation of a protein-ligand complex and enables calculation of the binding affinity [12]. sLe^x^ was docked to the human and mouse E-selectin structures using the AutoDock program with YAMBER3 force field. sLe^x^ was allowed full flexibility and E-selectin had a fixed backbone with flexible sidechains. 250 docking runs were completed, and the AutoDock scoring function sorted the runs by binding energy. Complex conformations were assumed to be different if the ligand RMSD was greater than 5 Å. Of the final conformations with positive binding energy, those for which there was no contact (5 Å or less) between the fucose residue of sLe^x^ and the calcium ion were eliminated as they would not be physiologically realistic. The docked complexes were solvated using the same MD steps as before with a water box of size 100 × 100 × 100 Å. The distance from the ligand to the calcium ion was analyzed over the simulation, and if it remained relatively constant, the complex was considered stable. The average free dynamics complex structures were used for the subsequent dissociation steps.

### SMD to simulate dissociation under applied force

SMD was used to simulate dissociation under applied force. Constant acceleration was applied to the ligand center of mass to move it away from the receptor center of mass. The simulations were run until all the hydrogen bonds between sLe^x^ and E-selectin broke and the two proteins dissociated.

### Microtube functionalization

Microrenathane tubes (300 μm i.d. and 50 cm long; Braintree Scientific, Braintree, MA) were sterilized with 75 % ethanol for 15 min. After three washes with PBS, the inner luminal surface was functionalized with recombinant human E-selectin (5 μg/mL) by incubating for 2 h, to allow for passive adsorption to the surface. Next, the microtubes were then incubated with dry milk powder (5 % w/v) in PBS for 1 h to prevent nonspecific adhesion. For control experiments, microtubes were prepared as indicated above except that E-selectin was replaced with BSA.

### Microsphere functionalization

SuperAvidin-coated microspheres (9.94 μm diameter; CP01N, Bangs Laboratories, Fishers, ID) were washed with PBS buffer per manufacture instruction. Next, the microspheres were incubated with Sialyl-Lewis^X^-biotin at specified concentrations for 1 h with gentle mixing every 15 min. Finally, the microspheres were washed twice and resuspended in flow buffer (PBS supplemented with 2 mM Ca^2+^). The surface density of sLe^x^ on the microspheres was not measured in this study, however our previous work with similar sLe^x^-coated microspheres and selectin surface coatings show that these materials recreate the physiological rolling behavior of leukocytes in the vasculature, with comparable rolling velocities [14].

### Rolling experiment

Functionalized microspheres (2x10^6^/mL) suspended in flow buffer were perfused through the microtubes using a syringe pump at 8 dyne/cm^2^. Recorded videos of rolling microbeads were captured and analyzed using ImageJ similarly to prior publications [15, 16].

## Results

### Mouse E-selectin homology model exhibits a greater interdomain angle than human E-selectin

**Fig. 2:**
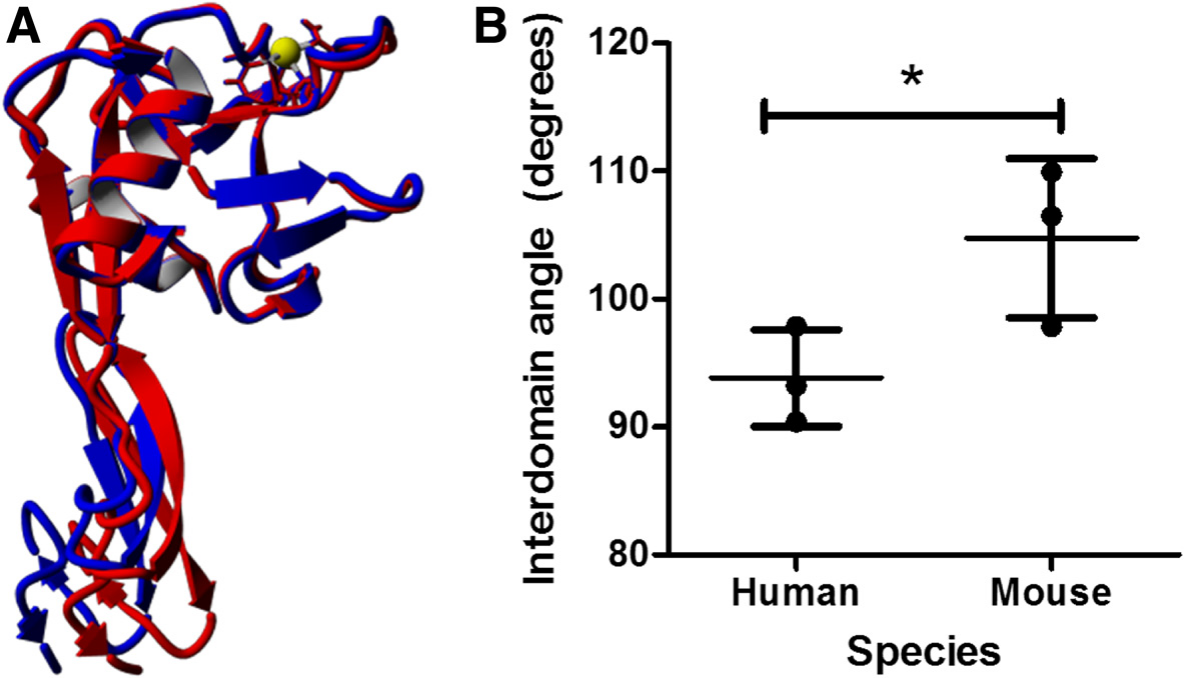
Mouse E-selectin showed a greater interdomain angle than human. **a** Mouse E-selectin is shown in blue and human E-selectin is shown in red. **b** The angle is measured from geometric center of residues 1–118 to the geometric center of residues 119–157 with a hinge at the pivot. Mean and standard deviation are plotted. Calcium ion is shown in yellow. *P value < 0.05 (two-tailed *t*-test)

Human and mouse E-selectin structures were solvated and equilibrated over the course of 10-ns MD simulations. Three simulations were performed for each species; the average structures for each species over the MD simulations were examined and compared. The most prominent structural difference between the two species was the interdomain angle between the EGF and lectin geometric centers. The mean interdomain angle for human E-selectin was 93.8° and the mean for mouse E-selectin was 104.8°, a difference of 11°. Fig. 2a shows overlaid representative human and mouse structures, and Fig. 2b shows the interdomain angle quantification.

**Fig. 3:**
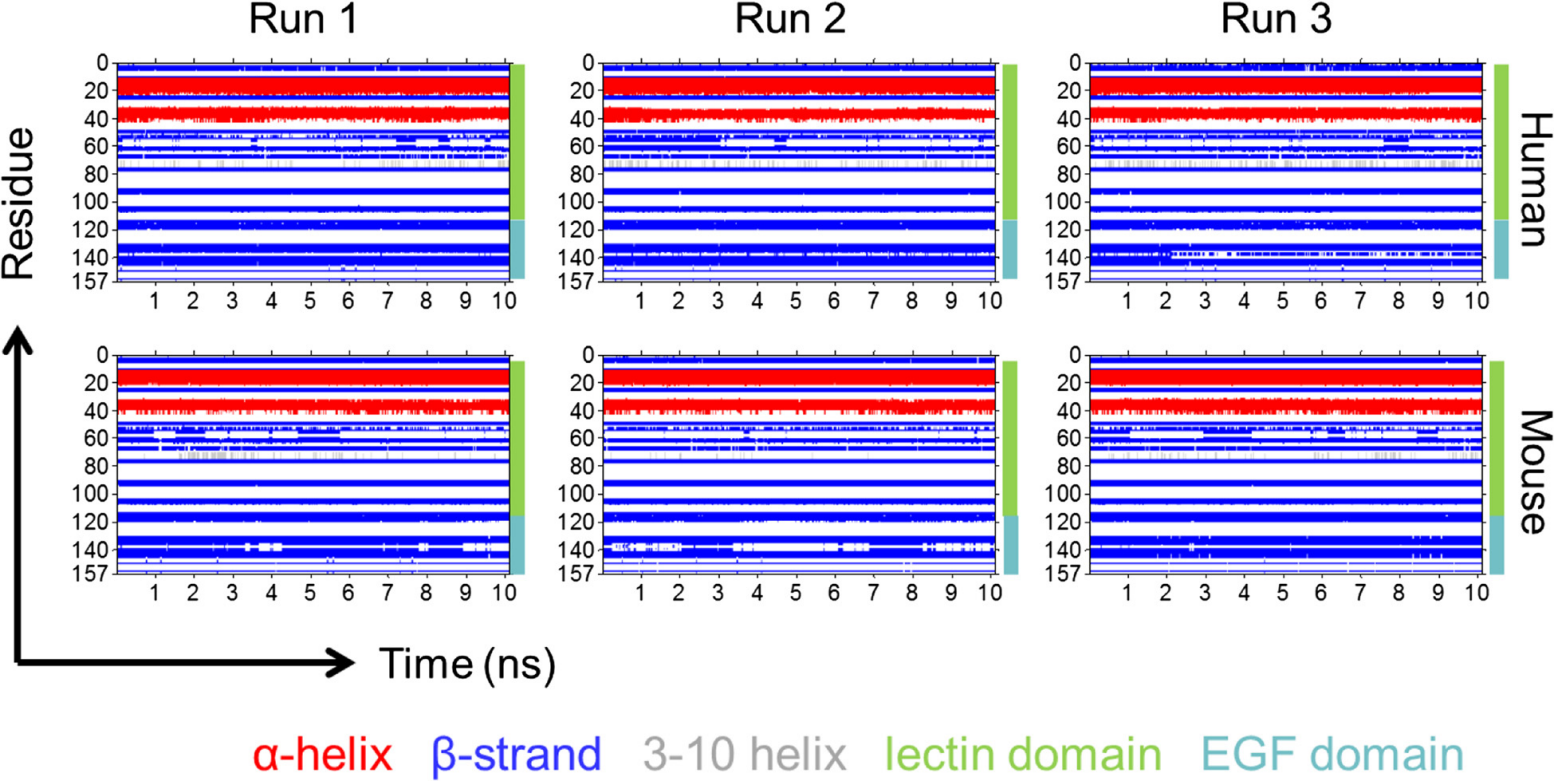
Dynamic secondary structure by residue of human and mouse E-selectin over 10 ns MD simulations. Residues are labeled by secondary structure according to their color: α-helices are red, β-strands are blue, 3–10 helices are grey, and coils and turns are not colored. The lectin domain includes residues 1–118, and the EGF domain is 119–157

Figure 3 shows the dynamic secondary structure by residue of each simulation run. The lectin domain for each species contains two α-helices: the C-terminal end of the first α-helix is shorter by one or two residues for mouse E-selectin, and both species show some fluctuation, known as “fraying” [17], in the length of the second α-helix, particularly on the C-terminal end. The β-strands in the remainder of the lectin domain vary in length for both species. In the EGF domain, the main structural features are two antiparallel β-strands. For the human runs 1 and 2, the beta-strands show little change in their length. In the human run 3, the two β-strands became fragmented into three after 2 ns. For the mouse, the β-strands show some variation in length for runs 1 and 2 but remain mostly stable for run 3. Overall, the mouse E-selectin lectin and EGF domains contains more random coil and turns than human E-selectin.

**Fig. 4:**
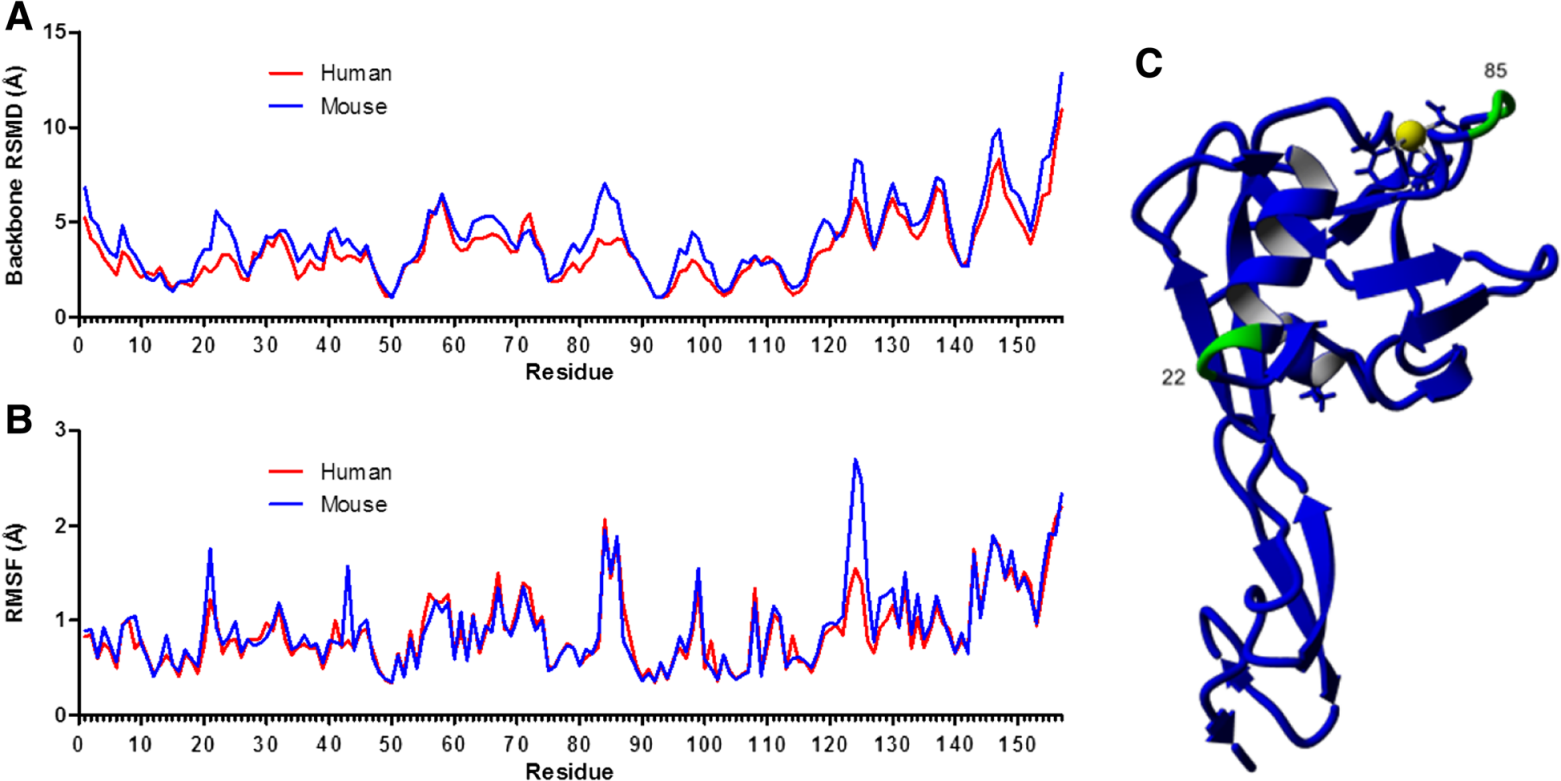
Residue differences between human and mouse E-selectin. Average backbone RMSD by residue (**a**) and average RMSF by residue (**b**) of human and mouse E-selectin during 10 ns MD simulations. **c** Mouse structure showing locations of residues 22 near the domain interface and 85 near the binding pocket

Looking more specifically at the residue differences between species, the average backbone root mean square deviation (RMSD) by residue was compared (Fig. 4a). Mouse E-selectin exhibited a greater backbone RMSD across nearly all residues. Specifically, the regions 1–3, 6–8, 21–25, 41–42, 64–66, 79–87, 96–100, 118–121, 124–126, 139, 145–151, and 153–157 showed a difference of more than 1 Å. Each of these regions contains amino acid differences between species. Importantly, many of these regions are involved with the pivot point between the lectin and EGF domains [18]. The flexibility of each residue was compared between species by examining the root mean square fluctuation (RMSF). Fig. 4b shows the RMSF by residue for each species, averaged over the three runs. The RMSF by residue was nearly similar between human and mouse, but the mouse shows peaks at residues 21, 43, and 124 whereas the human protein does not. As expected, these are all locations where there are one or more amino acid differences between species and all are locations of increased backbone RMSD (see Fig. 4a). Residue 21 and 43 are at the C-terminal end of the first and second α-helices, respectively. As shown in Fig. 2, the length of both α-helices fluctuated over the equilibration MD simulation. Residue 124 shows the greatest increase in RMSF and is located in a section of turns and coils in the EGF domain that is roughly parallel to the main β-strands. Figure 4c shows the locations of two residues where there was the greatest difference in RSMD for the mouse E-selectin. Residue 22 is located very close to the lectin/EGF domain interface, and residue 85 is close the binding pocket in the lectin domain.

### Mouse E-selectin is predicted to bind more strongly to sLe^x^ than human E-selectin

**Fig. 5:**
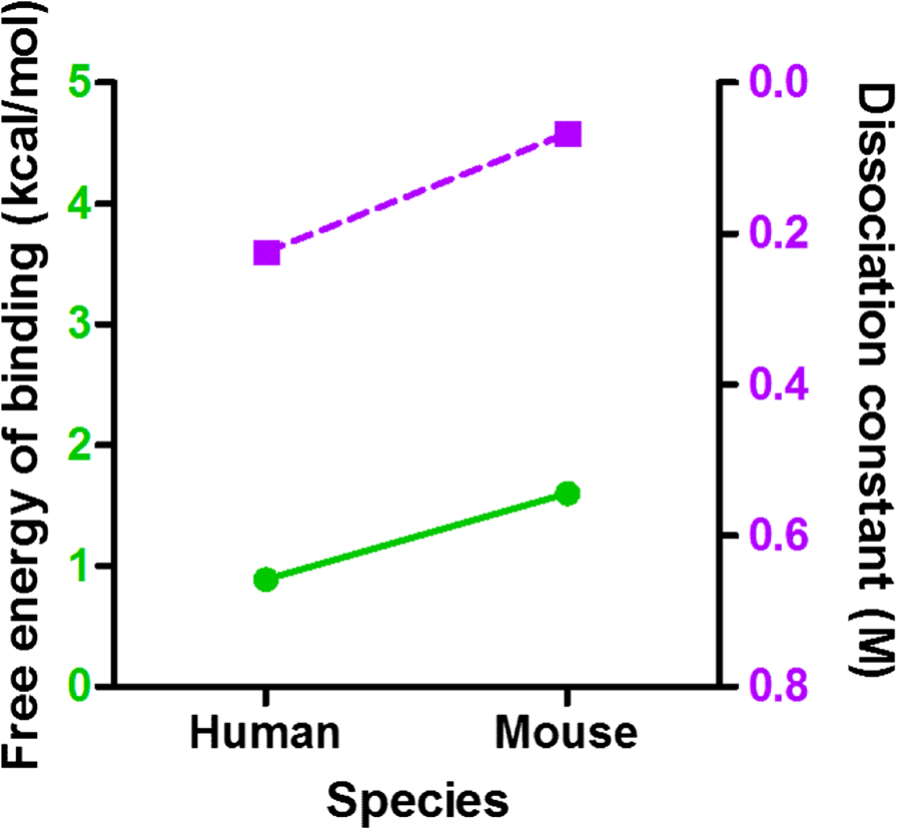
Free energies of binding and dissociation constants for human and mouse E-selectin:sLe^x^ complexes. Free energies of binding are shown with green circles and a solid line and dissociation constants are shown with purple squares and a dashed line

Equilibrated sLe^x^ was then docked onto the human and mouse E-selectin structures. The free energy of binding and the dissociation constant were ranked for each of the resulting complexes. Only stable complexes for which there was interaction with the calcium ion were considered [19], resulting in four feasible complexes for each species, and the highest free energy complex of each species was chosen for further study [20]. The mouse E-selectin complex yielded a higher free energy of binding as well as a lower dissociation constant (Fig. 5).

### Differences in dissociation time among complexes are caused more by interdomain flexibility rather than by contacts between receptor and ligand

**Fig. 6:**
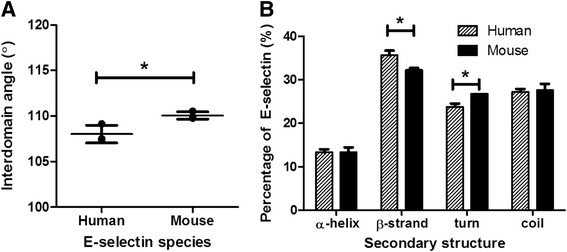
Differences in domain angle and secondary structure composition between species. **a** Angle between geometric center of residues 1–118 and geometric center of residues 119–157 for human and mouse complex configurations. **b** Secondary structure composition of E-selectin by species. Mean and standard deviation are shown. *P < 0.05 (two-tailed *t*-test)

The complexes were solvated and equilibrated for 10 ns. The average equilibrated complexes were examined prior to dissociation as per other studies of selectin binding [18, 21]. The geometric parameters analyzed included the distance and angle between the lectin and EGF domain centers of mass, the number of interdomain contacts and hydrogen bonds, the hinge distance, and the number of contacts and hydrogen bonds between the ligand and the receptor. Contacts were defined as less than 5 Å distance between two residues. As shown in Fig. 6a, the mean interdomain angle for the mouse-sLe^x^ complex was higher than for the human-sLe^x^ complex. Increased interdomain angle has been shown to increase flow-enhanced tether rate for N138G L-selectin [22], so it is predicted that mouse E-selectin will have a greater tether rate than human E-selectin. The secondary structure composition of both E-selectin species was examined (Fig. 6b). There was no significant difference in the percentage of α-helices and coil between species. However, mouse E-selectin in complex had a smaller percentage of β-strands and an increased percentage of turns compared with human E-selectin.

**Fig. 7:**
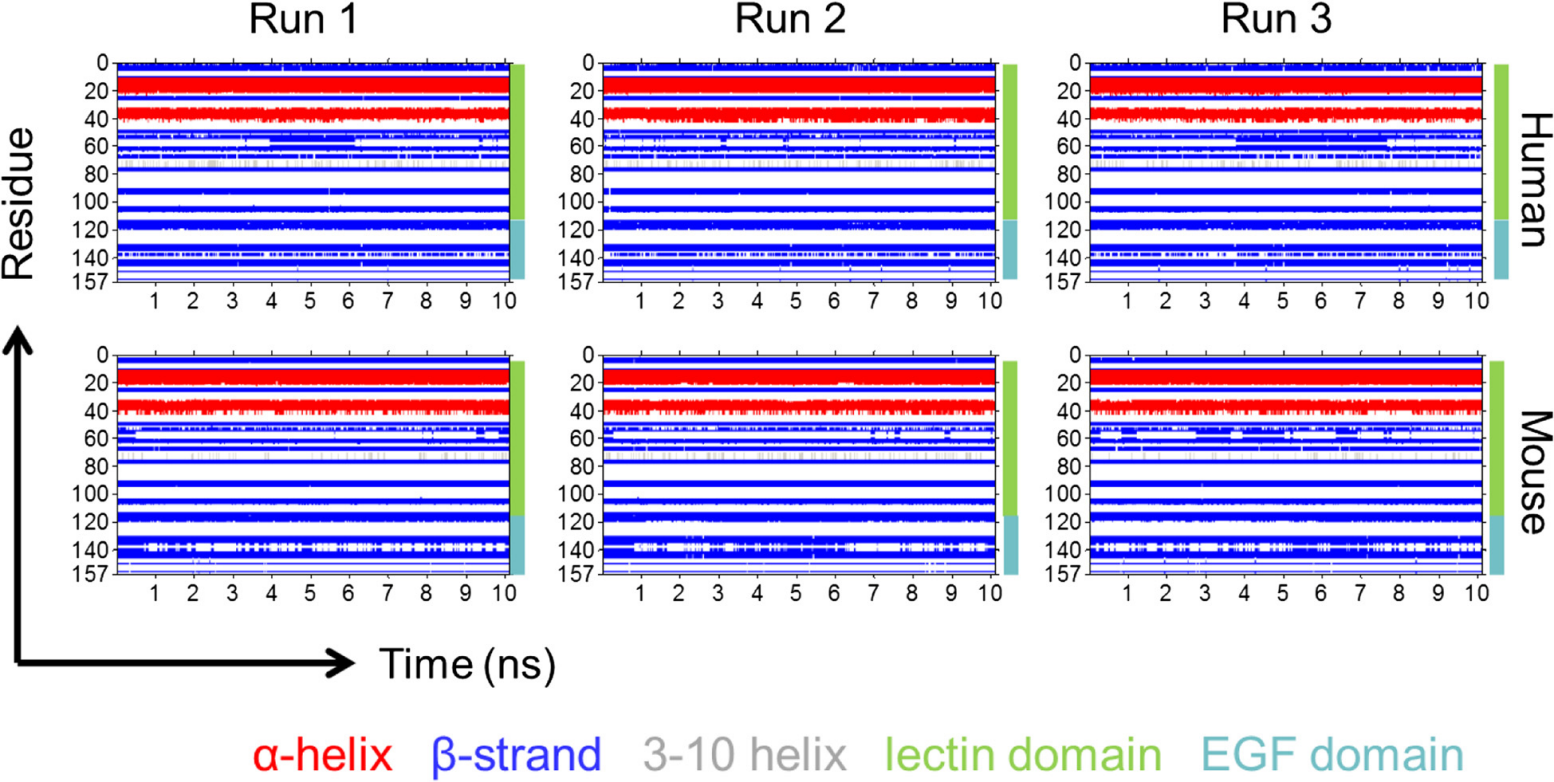
Dynamic secondary structure by residue of human and mouse E-selectin complexes over 10 ns MD simulations. Residues are labeled by secondary structure according to their color: α-helices are red, β-strands are blue, 3–10 helices are grey, and coils and turns are not colored. The lectin domain includes residues 1–118, and the EGF domain is 119–157

The secondary structure of each complex was examined over the solvated free dynamics simulation (Fig. 7). There was a notable difference in the antiparallel β-strands of the EGF domain between species. The mouse complex showed two such β-strands during each individual run and the length between the strands varied. However, all of the human complex runs oscillated between two or three short β-strands. For both species, the two α-helices in the lectin domain showed some fluctuation in the length, particularly on the C-terminal end of the second α-helix; this is similar to the trajectories of E-selectin alone (Fig. 2).

**Fig. 8:**
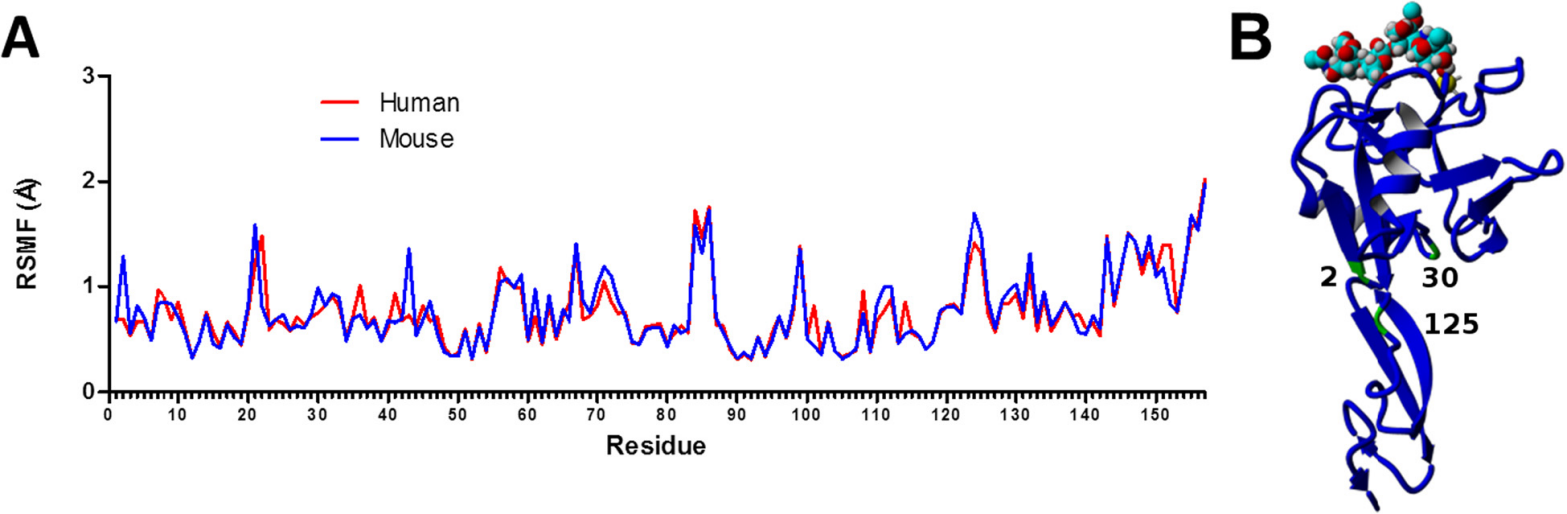
Interdomain hinge differences between species. **a** RMSF by residue for human mouse configuration complexes. **b** Mouse structure showing locations of residues 2, 30, and 125 near the domain interface

The residue flexibility of each species complex was examined by studying average RMSF values over the 10-ns free dynamics (Fig. 8a). Comparing the two species, the mouse complex exhibited a higher RMSF at several key pivot residues, including 2, 30, and 125 (Fig. 8b). There is also an RMSF peak at residue 43, which is at the C-terminal end of the second α-helix. Adhesion is largely regulated by the interdomain hinge, so increased flexibility in this area could indicate a prolonged bond lifespan and lower off-rate [8].

**Fig. 9:**
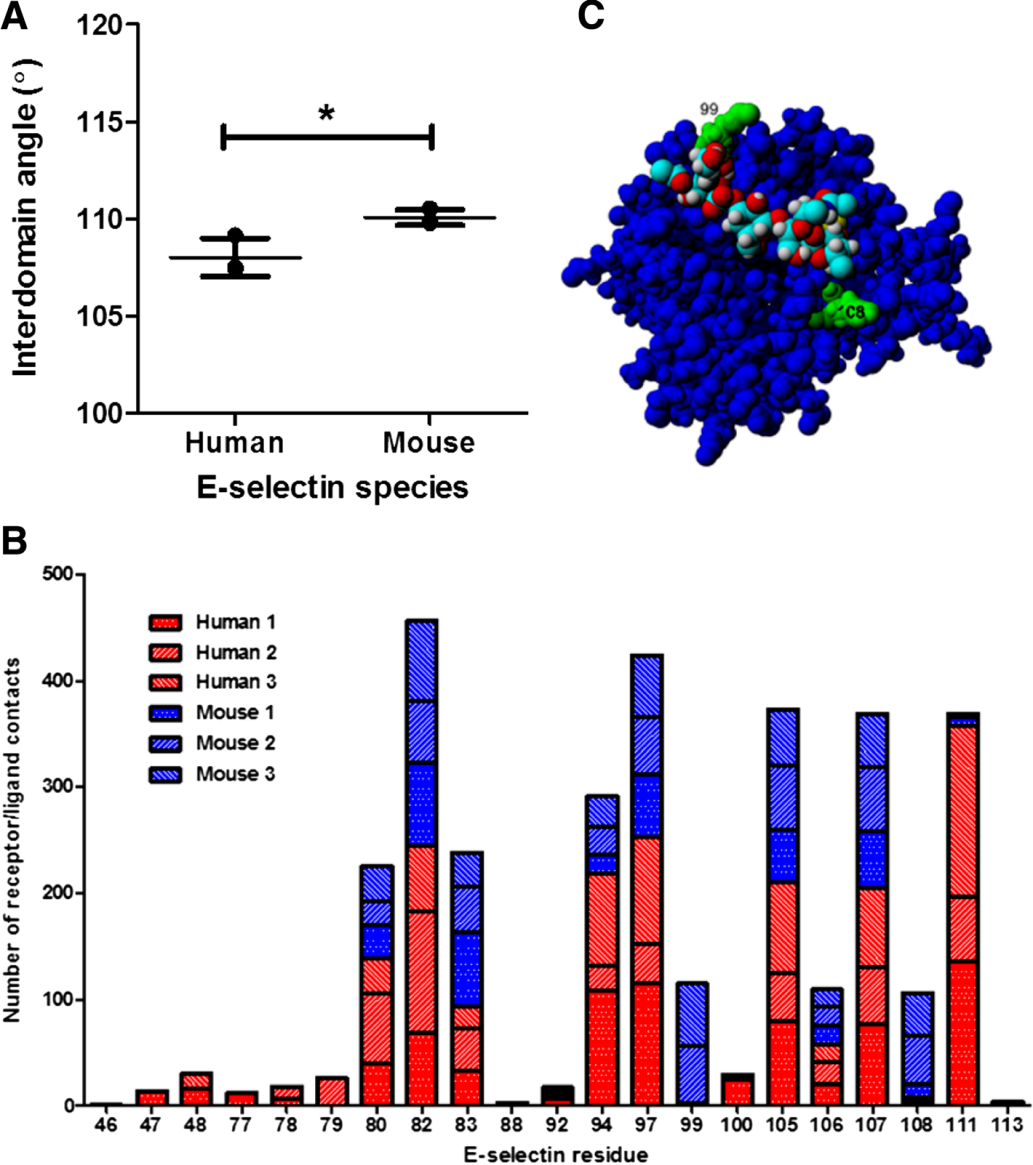
Receptor/ligand interface differences between human and mouse complexes. **a** Number of contacts between ligand and receptor within 5 Å. Mean and standard deviation are shown. *P < 0.05 (two-tailed *t*-test). **b** Distribution and quantification of receptor/ligand contacts for E-selectin residues that are within 5 Å of sLe^x^ for each human and mouse complex. **c** Mouse E-selectin looking down on lectin domain, showing locations of residues 99 and 108 relative to sLe^x^

The E-selectin residues in contact with sLe^x^ were examined for the average solvated 1G1T structure and human and mouse configuration complexes (Fig. 9). The human complex exhibited more contacts with sLe^x^, defined as the number of atoms of E-selectin that were within 5 Å of any atoms of sLe^x^. (Fig. 9a). The specific residues and number of contacts for each complex are shown in Fig. 9b. All of the E-selectin residues except residue 99 had RMSF values within 1 Å (Fig. 8a), indicating relatively low flexibility. This is consistent with their location within or near the binding site. Residue 82 had the most contacts, with residues 97, 105, 107, and 111 showing the next highest number of contacts. There were several contacting residues in the human complexes that had no or negligible contact for the mouse complexes, including 47, 48, 77, 78, 79, and 100. All of these residues had fewer than 50 contacts among the three runs. Conversely, two residues for which there was significantly more contact for mouse complexes than for human were 99 and 108 (Fig. 9c). Both residues 99 and 108 experienced about 100 contacts between the three mouse complexes; they are located on either end of the sLex and may serve as anchor points. Thus, despite having fewer total contacts and a similar number of residues in contact with sLe^x^, the data suggest that residues 99 and 108 are of particular importance in dissociation. Residue 99 is lysine and residue 108 is arginine, both large and positively-charged amino acids. Neither of these are residues that are different between human and mouse E-selectin but both are one or two residues away from substitutions at 98, 101, and 110.

### Mouse E-selectin complex takes longer to dissociate than human E-selectin

**Fig. 10:**
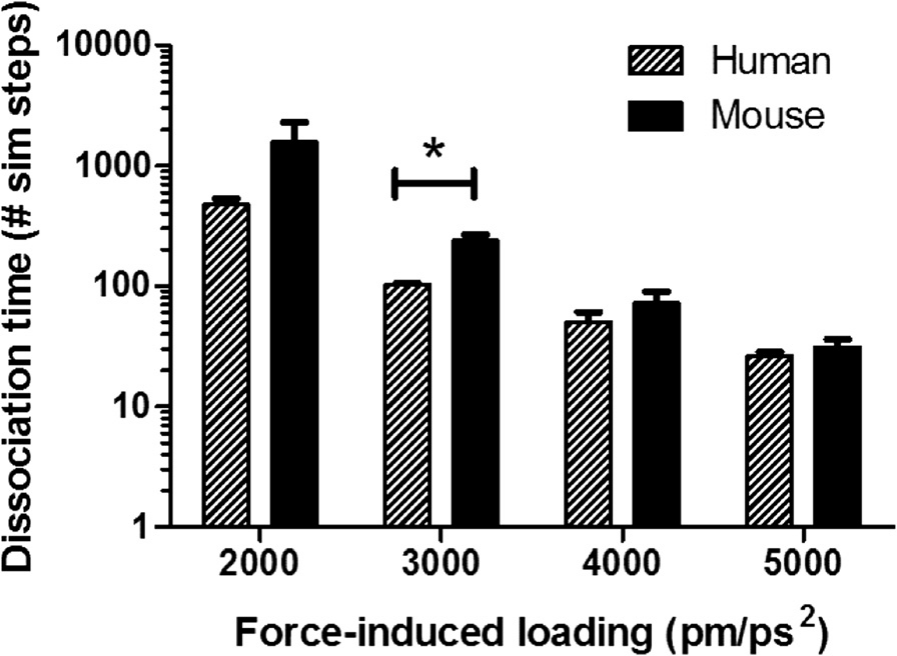
Dissociation time for mouse:sLe^x^ and human:sLe^x^ complexes at varying force-induced loading rates. Mean and standard deviation are shown. *P < 0.05 (two-tailed *t*-test)

Each species complex was subjected to force loading rates between 2000 and 5000 pm/ps^2^, and dissociation was determined as the point when all hydrogen bonds between the ligand and receptor were broken and did not reform. In all simulations, higher force-induced loading rates led to faster dissociation times (Fig. 10). Under all force-induced loading rates, mouse complexes took longer on average to dissociate. However, only the rate of 3000 pm/ps^2^ led to a statistically significant difference between species.

**Fig. 11:**
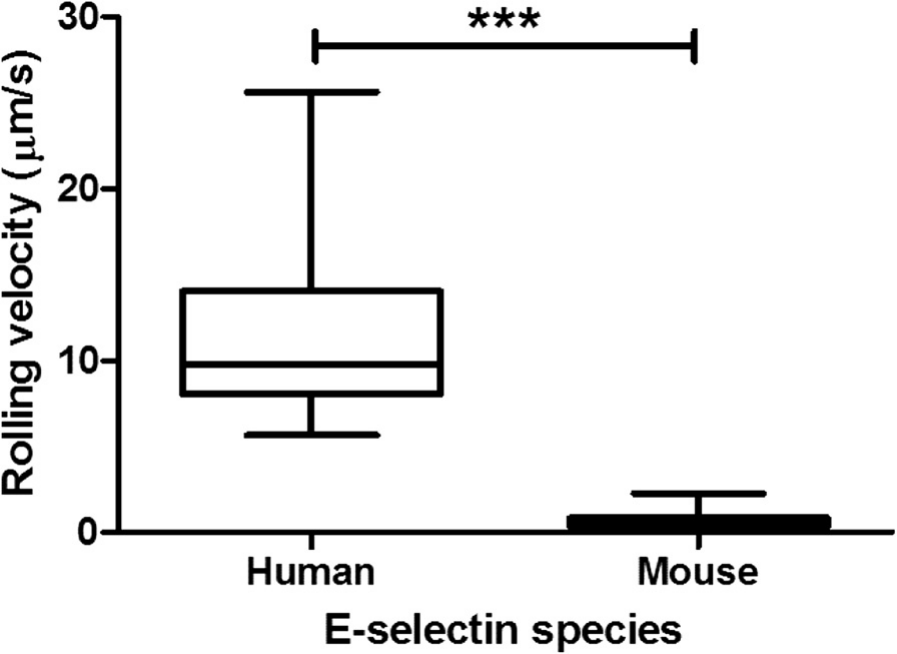
Rolling velocity of sLe^x^-coated microspheres perfused through an E-selectin coated microtube. Mean and standard deviation shown. ***P < 0.001 (two-tailed *t*-test)

sLe^x^-coated microspheres were perfused through E-selectin coated microtubes and the average rolling velocity of the microspheres on each E-selectin species were compared (Fig. 11). Microspheres were used instead of cells to eliminate effects of cell deformability or other selectin:ligand pairs not considered within the scope of this study. As expected, the microspheres rolling on mouse E-selectin showed a statistically significantly lower rolling velocity compared to microspheres perfused over human E-selectin; the average rolling velocity on human E-selectin was 11.2 μm/s and the average for mouse E-selectin was 0.63 μm/s. Rolling velocity is largely affected by off-rate [23], so the longer dissociation exhibited by simulations of the mouse E-selectin complex versus the human complex (Fig. 10) is consistent with this trend.

## Discussion

Excessive leukocyte extravasation out of the bloodstream has been linked with chronic inflammation [4]. Thus, potential therapies for controlling the inflammatory response could involve inhibiting or moderating the selectin adhesion that mediates leukocyte tethering and rolling to the blood vessel walls. Homology modeling and amino acid substitutions, particularly those that affect molecular flexibility, and have been shown to be highly effective in changing adhesion and inhibitive function [24–26]. In this study, a mouse homology model comprising 29 point substitutions to the human E-selectin crystal structure greatly affected dissociation of sLe^x^ from the resulting complex. The adhesive characteristics of the mouse E-selectin homology model qualitatively match results from experiments that showed slower rolling velocity of sLe^x^-coated microspheres. These results provide new insight into the connection between structure and function of species-specific E-selectin. These results suggest that differences in dissociation time result more from interdomain flexibility than by contacts between receptor and ligand.

Docking a homology model structure does accumulate more errors than using a crystal structure [27], but in this case, a crystal structure for mouse E-selectin was not available. The docking algorithm accounts for two important details: protein flexibility is a key determinant in binding, and physiologically, complexes are solvated in a salt solution [19]. The docking algorithm included flexibility in the E-selectin side chains and full flexibility in the sLe^x^. The docked structures were solvated after docking using 10-ns MD simulations to allow for more physiological conditions. Intramolecular distortion of the lectin and EGF domains was not evident for most simulations, particularly at higher force-induced loading rates. It has been shown that shear flow can have a contribution to intramolecular distortion [21], but as with most selectin:ligand dissociation simulations [13], shear flow is not directly considered in these SMD simulations.

This study demonstrates the significance of combining simulations with experimental rolling studies to gain insights into the functional differences between proteins that share sequence similarity. The differences in amino acid structure can be exploited for applications such as selectin-based leukocyte and circulation tumor cell isolation [28]. The combined methodology involving docking, SMD, and MD simulations of receptor:ligand interactions holds possibility as a means for rational drug design [29].

## Conclusions

Molecular simulations were used to elucidate the binding of sLe^x^ to mouse and human E-selectin. Docking simulations predicted that mouse E-selectin would bind more strongly to sLe^x^ than human E-selectin, and SMD simulations predicted that the mouse E-selectin:sLe^x^ complex would exhibit a longer dissociation time. Mouse E-selectin alone and bound to sLe^x^ exhibited a greater interdomain angle than human E-selectin, and there were fewer receptor:ligand contacts. When tested experimentally, sLe^x^-coated microspheres rolled more slowly in tubes coated with mouse E-selectin rather than human E-selectin.

## Abbreviations

BSA, bovine serum albumin; EGF, epidermal growth factor; MD, molecular dynamics; PBS, phosphate-buffered saline; PDB, protein data bank; RMSD, root mean square deviation; RMSF, root mean square fluctuation; sLe^x^, sialyl Lewis x; SMD, steered molecular dynamics
